# Assessing Seedbank Longevity and Seed Persistence of the Invasive Tussock Grass *Nassella trichotoma* Using in-Field Burial and Laboratory-Controlled Ageing

**DOI:** 10.3390/plants11182377

**Published:** 2022-09-12

**Authors:** Talia Humphries, Singarayer Florentine

**Affiliations:** The Future Regions Research Centre, School of Science, Psychology and Sport, Federation University Australia, Mount Helen, VIC 3350, Australia

**Keywords:** invasive species, seed ecology, seed longevity, seedbank persistence, invasive species management, *Nassella trichotoma*

## Abstract

The ability to produce highly dense and persistent seedbanks is a major contributor to the successful widespread establishment of invasive plants. This study seeks to identify seed persistence and seedbank longevity for the invasive tussock grass *Nassella trichotoma* (Nees.) Hack. ex Arechav in order to recommend management strategies for preventing re-emergence from the seedbank. To determine the seedbank longevity and persistence, two experiments were conducted: (i) seeds were buried at four depths (0, 1, 2, and 4 cm) and collected and assessed for viability, seed decay, and in-field germination after 6, 9, 12, 15, and 18 months of field burial; and (ii) seeds were exposed to artificial ageing conditions (60% RH and 45 °C) for 1, 2, 5, 9, 20, 30, 50, 75, 100, and 120 days, and viability was determined through germination tests and tetrazolium tests. Less than 10% of the seeds collected after 12 months of in-field burial were viable. The artificial ageing treatment found germination declined to 50% after 5.8 days, further suggesting that *N. trichotoma* seeds are short lived. The results from both experiments indicate that *N. trichotoma* has a transient seedbank, with less than 10% of the seeds demonstrating short-term persistence. It is likely the persistent seeds beyond 12 months were exhibiting secondary dormancy as viable seeds did not germinate under optimal germination conditions. The “Best Practice Guidelines” recommend monitoring for seedbank recruitment for at least three years after treating *N. trichotoma* infestations. The results of this study support this recommendation as a small proportion of the seeds demonstrated short-term persistence.

## 1. Introduction

Human interactions with many grassland ecosystems have led to the significant alteration of their natural state, and as a consequence, there is a severe threat of ongoing degradation to grasslands ecosystems in many parts of the world [1]. As an example, the Greater Western Plains, located in central Victoria, Australia, provides a case illustration of the arising degrading pressures acting on these endangered grasslands, including: increased grazing regimes, habitat fragmentation, increased urban expansion, and the introduction and widespread establishment of invasive plant species [2,3]. To date, approximately 1% of the once extensive Greater Western Plains remains in its historic condition [4]. It is now understood that for ecosystems which have been reduced to a degraded state, conservation and restoration efforts must be prioritised and implemented in order to assist them in remaining resilient in the face of changing climate conditions and projected increases in natural disasters [5]. In this regard, conservation and restoration efforts are often thwarted by the presence of invasive plant species, and, of key importance to this investigation, while efforts to reduce the aboveground cover of significant weeds is regularly implemented, efforts to control their seedbanks are often overlooked.

In terms of degraded grasslands, the presence of large, persistent seedbanks of invasive species is a key indicator of the precarious state of an ecosystem [6]. When these invasive seedbanks are high in density, they have a significant competitive advantage over native species, since (i) they physically displace germinating species in the search for water, nutrients, and light, and (ii) they can alter the biotic and abiotic composition of the ecosystem in favour of the invasive species [7]. Some work on controlling the seedbank of a weed-dominated site has demonstrated that this action improves restoration success [8,9], and it has been suggested that restoration efforts that seek to understand seedbank density and persistence of the dominant invasive species can significantly assist in the ongoing management of degraded areas [10].

Understanding the seedbank dynamics of invasive species can uncover important ecological factors that can better equip land managers with information for developing effective and sustainable control strategies [11,12]. Therefore, research that provides a greater understanding of an invasive species’ seedbank longevity and persistence should be prioritised [10,12,13,14]. Although these two factors are similar, seedbank longevity refers to the length of time a seed can remain viable and germinate successfully [15], whereas seedbank persistence refers to the ability of a seed to resist various environmental pressures before losing viability [16]. Seed longevity studies are often conducted ex situ, where the seeds are exposed to elevated humidity and temperature to rapidly age the seeds [17,18,19]. This process of artificial ageing testing for seed vigour and longevity provides rapid results, and this can provide insight for understanding issues such as invasive potential, soil seedbank eradication programs, and future monitoring in a short timeframe [10]. Seed persistence studies are required to be conducted in situ, where seeds are buried to observe the effects of different seasons, climates, duration of burial, or burial depths on seed viability [20,21]. Seed burial trials are useful for identifying how real-world factors such as burial depth, soil moisture, and pathogens influence seed longevity [22,23]. Additionally, the proportion of seeds that germinate under field conditions can be observed [24]. The comparison of these two methods may provide enhanced insight for identifying the seedbank longevity and persistence of an internationally significant, aggressive, invasive grass species: *Nassella trichotoma* (Nees.) Hack. ex Arechav. (known commonly as serrated tussock) of the Poaceae family [17]. Wide variations in the seed longevity for this species have been reported in the literature, with claims of seeds aged between 1 to 13 years germinating successfully under soil conditions [25,26]. It is widely reported in publicly available management material that this species’ seedbank can persist for up to three years [26].

It has been determined that seed longevity is related to the availability of stored macromolecules such as nucleic acids, lipids, and proteins together with their ability to protect and repair these resources under stressful conditions [27]. The current categorisation of seedbank resilience refers to (i) transient seedbanks, which do not persist in the soil for more than one year, (ii) short-term persistent seedbanks, which can remain viable from one to five years, and (iii) long-term persistent seedbanks, which are viable for at least five years [28]. Notwithstanding the attraction of this somewhat simple framework, observed persistence varies greatly between species and biotypes, and is affected by different environmental cues and disturbances, which may differ from year to year [7,21]; although the development of a simple categorisation system for the seedbank of an invasive species is a powerful and rapid management tool, it overlooks the various environmental pressures that alter seedbank persistence.

The position of the seed within the seedbank can alter its experienced microhabitat, significantly influencing the seed’s persistence [21]. A common example of this microhabitat trend is that shallow burial depth and increased interred duration result in a significant decline in seed viability, as seen in *Andropogon gayanus* [29], *Raphanus raphanistrum* [30], *Erigeron canadensis* [31], and *Artemisia tridentate* [14]. In this regard, seeds located on or just below the soil surface are exposed to more intense fluctuations in soil moisture and temperature compared to deeper buried seeds. These fluctuations can result in shallow covered seeds experiencing more intense wet and dry cycles, as was observed in *A*. *tridentate,* where it was found that after two years only 0-11% of viable seeds remained at the soil surface compared with almost half of the seeds maintaining viability at a 3cm depth [14]. Seed predation is also increased at shallow depths [32]. Due to these factors, it has been observed that seeds that are buried deeper into the soil profile are better protected from a range of devitalising disturbances and persist in the seedbank for a longer period of time compared to those buried closer to the surface [14,29]. The trade-off, however, is that at increased depths, seed dormancy is usually prolonged, particularly for photoblastic seeds [33], leading to observations that with increased depth, successful emergence is reduced, particularly in smaller seeds with less energy reserves [34].

One of the most aggressive and difficult to manage weeds in the Greater Western Plain’s grasslands is *N. trichotoma*. This South American native is considered a significant weed in southeast Australia [35,36], New Zealand [37], and South Africa [38]. It is also problematic within its native range due to its low palatability for grazing animals and livestock [39,40]. Individuals of this species can produce over 100,000 anemochory seeds annually, which are dispersed within the panicle up to 20km from the parent plant [41]. As a consequence of the high fecundity, seedbank densities of up to 50,000 seeds per square metre have been recorded [38,40], and rapid germination has been observed following disturbance events and heavy rainfall [41].

The majority of the seeds remain within the top 2.5 cm of the soil layer [38] and the optimal emergence occurs within this depth, whereas the emergence of seedlings buried at 4 cm was significant reduced [42]. The rounded plump seeds are 3 mm in length when measured from the hooked tip to the base, contain an off-centred 25–35 mm long awn, and weigh 76–86 mg [42]. The seed drop begins in early summer when the panicles containing seeds droop over and detach from the plant, and subsequently, they are dispersed by strong winds. Under favourable conditions, the seed drop may begin as early as October and continue up until May [41,43]. Under favourable years, a single plant can produce up to 2000 panicles [44].

*Nassella trichotoma* has non-deep physiological dormancy, which is broken by increased rainfall in Autumn, and some research suggests that the seeds can initiate dormancy at any time of the year to avoid unfavourable growing conditions [26]. As seeds dropped late in the seeding period, for example, in late April or May, will miss the optimal germination cues provided in autumn, these seeds may contribute to extending the seedbank longevity for this weed [45]. The combining factors of uneven germination, a potentially long-lived seedbank, and a large annual seed set with wide dispersal make recruitment from the seedbank one of the most challenging aspects for managing this species and achieving grassland restoration goals.

This paper aims to integrate seedbank persistence and longevity methodologies to make confident recommendations for the management of *N. trichotoma’s* seedbank. With the wide variability in seedbank longevity reported for *N. trichotoma*, a study combining and comparing the results of in situ (seed burial) and ex situ (artificial ageing) techniques should provide greater clarity regarding the seedbank ecology for this species. Long et al. [17] compared the results of artificial ageing to field persistence data of 27 invasive plants, and found the two techniques to provide a positive correlation. The benefits of using both techniques allow for the consideration of the real-world factors on seed longevity; however, as these experiments are limited by time, the rapid ageing technique can assist in identifying any seeds that persist beyond the burial treatment timeframe.

## 2. Methods

### 2.1. Seed Collection and Preparation

Panicles containing seeds were collected from over 100 mature *N. trichotoma* plants in early December 2018. Seeds were collected from a *N. trichotoma*-dominated grassland in Mambourin, Victoria, Australia (37°55′18.12″ S, 144°32′43.079″ E). The panicles containing the seeds were sealed within a plastic zip-lock bag and transported to Federation University Australia, Mount Helen Campus, located approximately 100 km from the collection site, where they were stored in darkness at room temperature (approximately 20 °C) until being placed into nylon mesh bags approximately three months after collection. The mesh bags containing the seeds were stored within a drawer at the Federation University Australia’s seed ecology lab until they were required for the subsequent experiments. Prior to use, a germination test confirmed the average seed viability was 75.32%.

### 2.2. Seed Longevity

In order to assess the longevity of *N. trichotoma’s* seedbank, the artificial ageing technique, developed by the Millennium Seed Bank Project, Royal Botanical Gardens, Kew, UK [18], was implemented. To prepare for this treatment, 45 plump, viable seeds (viability was determined by gently squeezing the seeds with forceps) were placed into bags made of fine (0.05 mm), semipermeable nylon mesh, which were sealed using hot glue. For the rehydration process, the mesh bags containing seeds were placed on a stand above a 47% relative humidity (RH) lithium chloride (LiCl) solution (370 g/L deionised H_2_O) within a sealed electrical box (300 × 300 × 102 mm), which was then placed in an incubator set to 20 °C and 24 h darkness for 14 days. After this time, the seed eRH was assessed using a hygrometer (HygroPalm HP23-A/HP23-AW-A handheld indicator, Rotronic) and considered successful with a reading of 47%. After the seeds were considered rehydrated, the seed bags were moved to a second electrical box set to 60% RH (LiCl, 300 g/L) and placed in an incubator set to 45 °C for the ageing treatment. At each sampling period, six seed bags were randomly selected and removed from the ageing box on days 1, 2, 5, 9, 20, 30, 50, 75, 100, and 120. At each sampling period, 25 seeds were randomly selected from each of the six replicates to undergo a standard germination test. For the germination test, the selected seeds were plated into Petri dishes lined with a single layer of sterilised Whatman 1 No. 10 filter paper and then moistened with 10 mL of sterilised RO water. The Petri dishes were sealed with parafilm and placed into an incubator (Thermoline Scientific, temperature and humidity cabinet, model: TRISLH-495–1-s.d., Vol. 240, Sydney, Australia), which was fitted with cool-white fluorescent lamps that provided a photosynthetic photon flux of 40 mmol m^−2^ s^−1^. The incubators maintained alternating temperatures of 25/15 °C, with 12 h of light and 12 h of darkness [42]. Seeds were observed for germination twice a week, and the seeds were incubated for 42 days, or until all seeds had successfully germinated. The remaining 20 seeds in each replicate were assessed for viability using only a tetrazolium test.

### 2.3. Seedbank Persistence

To identify the seedbank persistence of *N. trichotoma*, 50 viable seeds were placed into 5-centimetre square bags made of fine (0.05 mm), semipermeable nylon mesh that allowed for the natural flow of water and micropathogens while keeping the seeds contained. The mesh bags were sealed using hot glue. One bag was placed into a hole that was dug to a depth of either 0 (surface), 1, 2, or 4 cm, and then covered with the excavated soil.

**Figure 1:**
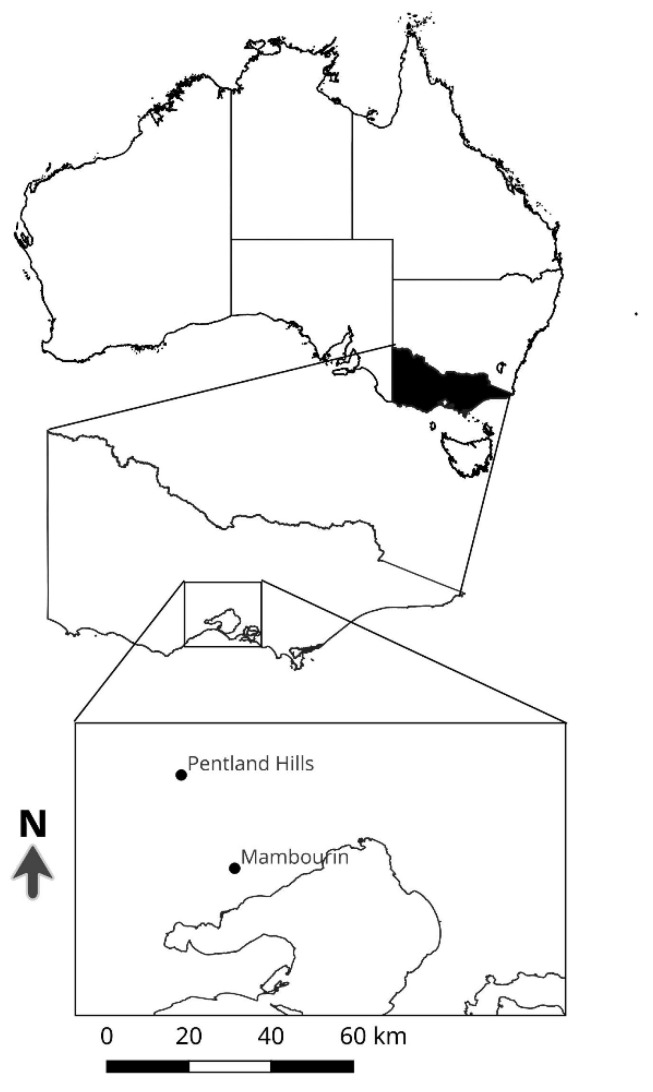
The location of the two sites used to investigate the seedbank persistence of *N. trichotoma*. The two sites are located within the Greater Western Plains grasslands, located to the west of Melbourne, Victoria.

As it was mentioned in the introduction, the seed set of *N. trichotoma* normally occurs in December for the southern hemisphere; however, in the case of climatically favourable seasons, seed set has been observed to continue throughout autumn. To determine if this time variation of the seed entering the soil seedbank influences the seedbank persistence, half of the seed bags were buried in January at Mambourin (37°55′18.12″ S, 144°32′43.079″ E), whilst the remainder were buried in May at a privately-owned site dominated by *N. trichotoma* that is located in the Pentland Hills, Victoria (37°39′50.515″ S, 144°24′10.334″ E) (Figure 1). The sites are located within 50 km of each other, and share similar rainfall and air temperature conditions.

At 6, 9, 12, 15, and 18 months after burial, five replicates from each depth were randomly exhumed from the soil and placed into labelled plastic zip-lock bags for transporting the mesh bags containing seeds to Federation University Australia’s seed ecology laboratory, located at Mt. Helen, Victoria, for examination. Seeds that germinated under field conditions (determined by visible radical emergence, intact root, or cotyledon) were recorded and discarded. The condition of the remaining seeds was assessed using the crush test, whereby gentle pressure is applied to the seeds by tweezers, and seeds that collapsed were deemed to have decayed [46]. Seeds that maintained their integrity were considered intact and were tested for viability via germination trials. These intact seeds had excess dirt removed using tap water and were assessed using the germination method previously described. At the completion of the 42-day incubation period, the remaining seeds were assessed by the crush test and any seeds that maintained their integrity underwent a tetrazolium test for viability.

### 2.4. Data Analysis

The effect of seed burial depth and duration on seed longevity of *N. trichotoma* was determined by transforming the data to an averaged percentage. The data were graphed on a cluster bar graph to show the effect of burial on time and burial depth on the longevity factors of viability, in-field germination, and seed degradation under field conditions.

For the artificial ageing treatment, a probit analysis to find the *P_50_* value was performed on the data using SPSS statistical software [46]. Seed viability was plotted against time (days) and a seed survival curve was fitted to the data. The number of final germinated seeds was converted into a percent of germination (%) [47]. The number of germinated seeds at 14 days of incubation was used to find the germination energy. Mean germination time was calculated using the equation:

Mean germination time = ΣDn/Σn (1)

where n is the number of seeds, which were germinated on day Dn, and n is the number of days counted from the beginning of germination [48].

The germination rate index was calculated as described by the Association of Official Seed Analysts [49] by using the following formula:

No. of germinated seeds/day of first count + … + No. of germinated seeds/day of final count.

## 3. Results

### 3.1. Seed Longevity

**Figure 2:**
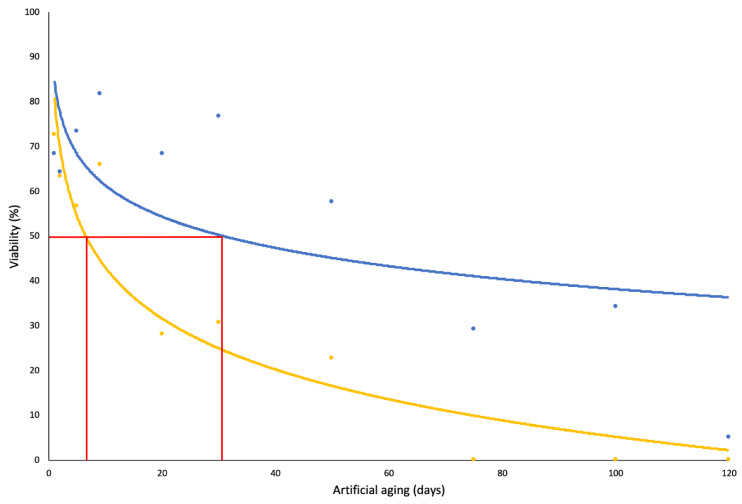
The effect of artificial ageing on viability (%) for *N. trichotoma* determined by germination (yellow) and tetrazolium (blue). Survival curve was fitted to each data set to represent the confidence limits of the probit analysis. The probit analysis determined *P_50_* = 5.8 days and *P_50_* = 31.3 days for the germination and tetrazolium trials, respectively, and these values are highlighted in red.

The effect of the artificial ageing experiment found that germination (%) steadily declined with increased exposure to the ageing treatment. The control viability (shown in the Section 2) determined germination (%) to be 75.35, and therefore, no significant variation was observed after one day of exposure to the ageing conditions. After two days of exposure to the ageing conditions, the germination (%) was reduced to 63.33%, and, with the exception of day nine, germination (%) continued to decline at each sampling period (Table 1). No germination was observed at or after 75 days of exposure to the ageing treatment. The seeds exposed to the ageing treatment for 1 and 9 days had higher germination energy compared to all other days (52.67% and 57.33%, respectively), which indicates over half of the seeds had germinated within the first 14 days of the germination trial. The seeds recovered at the same sampling periods were also observed to have a higher germination rate index (3.76 and 3.96, respectively) compared to those retrieved on day 5, 20, 30, and 50, and the germination rate for the latter three sampling periods was considerably low. With the exception of the seeds exposed to the ageing conditions for 50 days or more, the mean germination time remained steady throughout the treatments, with the average germination occurring at approximately 24 days of incubation. The germination trials achieved *P_50_* by 5.8 days, whereas this value increased to 31.2 days in the tetrazolium test for viability (Figure 2). The probit analysis for the germination trials suggests that approximately 10% of the seeds would survive at least 100 days of the artificial ageing treatment.

### 3.2. Seedbank Persistence

**Figure 3:**
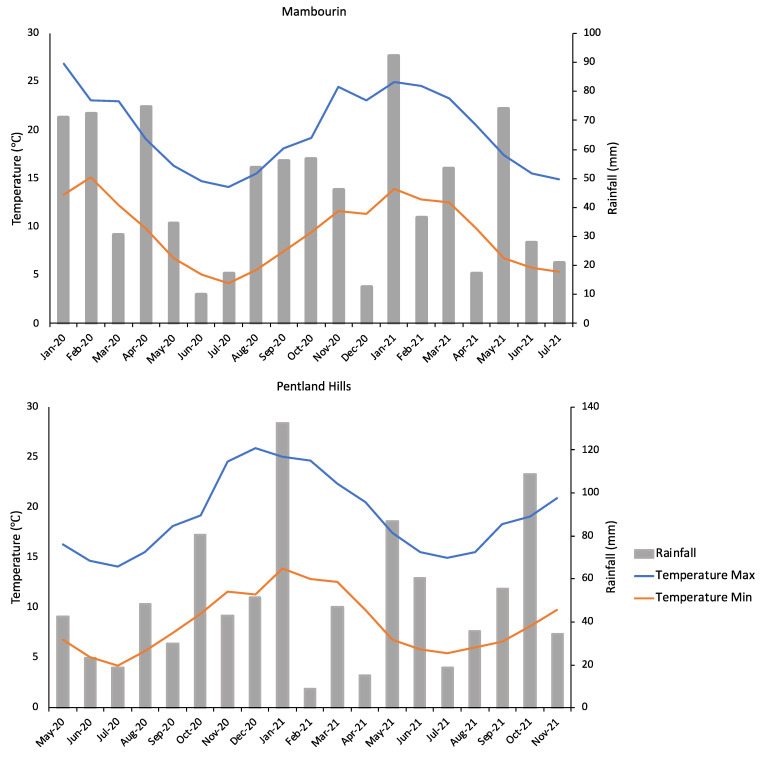
The climate data for Pentland Hills and Mambourin for the duration of the seed burial trials at each location. Data were accessed from the Bureau of Meteorology [50]. Temperature and rainfall data for the Mambourin site were taken from Mt. Rothwell weather station, which is located approximately 15 km from this site. The temperature data for Pentland Hills were collected from the Durdidwarrah weather station, located approximately 40 km from the site, and the rainfall data were collected from the Merrimu Reservoir weather station, located approximately 13 km from the Pentland Hills site.

The climate conditions, in terms of temperature and monthly rainfall, were similar at the two selected sites, Pentland Hills and Mambourin, for the duration of the burial trials (Figure 3). The sum of rainfall at Pentland Hills was higher (940 mm in total) than that observed at the Mambourin site (857 mm). Similar maximum temperatures were observed at both sites, with the average summer temperature reaching 25 °C at the Mambourin site and 24 °C at the Pentland Hills site, with both sites averaging a top temperature of 14 °C during the winter months. The Mambourin soil had a dark-brown colour and a clay to clay-loam texture, and the site was relatively flat in topography. The Pentland Hills site had soil with a loam texture, was dark reddish-brown in colour, and the seeds were buried on a slight slope close the top of the hill with a southern aspect. It is known that *N. trichotoma* can germinate under a wide range of temperatures, with a high germination (%) observed under alternating temperatures from 17/7 °C to 30/20 °C [42].

The depth of seed burial did not impact the seedbank persistence for *N. trichotoma*, as similar observations in viability were observed across all burial depths (Table 2). The seedbank persistence appeared to reduce as a result of an increased time of burial (duration) and the season the seeds were buried, with those buried in May exhibiting greater persistence than those buried in January. The proportion of seeds to have decayed under the field conditions was high across all the retrieval times for both the January and May burial times. It was observed that seed viability (%) decreased with an increased burial duration for the seeds buried in May, but this was not the case for the seeds buried in January. The highest proportion of viable seeds recovered (15.2%) were buried in May at 1cm for nine months. Overall, only a small proportion of the seeds were intact and viable at any of the sampling periods, suggesting that seed persistence rapidly declines in *N. trichotoma*.

## 4. Discussion

### 4.1. Seedbank Longevity

Understanding key biological traits of invasive species, such as seed longevity, is critical for developing effective management policies and strategies for long-term control and, potentially, local eradication [51]. The artificial ageing technique has proven to be an effective and rapid tool for determining if a species can produce transient, short- or long-term persistent seedbanks [10,17]. The artificial ageing treatment determined that *N. trichotoma* has a short-lived seedbank, with a *P_50_* value of 5.8 days. It was previously determined that a *P_50_* value < 20 days corresponds to short-lived, transient seedbanks [17,28]. The results of the seed longevity experiment are consistent with our findings in the seedbank persistence experiment, whereby 99% of the January seeds and 91% of the seeds buried in May had either germinated under field conditions or had decayed within the first year.

Germination energy refers to the ability for seeds to germinate fast and in unison, as the more seeds that germinate within a pre-defined timeframe can indicate the level of seed vitality [52,53]. The *N. trichotoma* seeds demonstrated decreased germination energy with increased exposure to the artificial ageing experiment. This can indicate that the emergence vigour of seedlings declines with increasing age [54]. There was little variation in the mean germination time for seeds exposed to the ageing conditions between 1 and 30 days; however, this time increased after 50 days of exposure. A higher mean germination time was observed to reduce the mean emergence time of subsequent seedlings [55], which further supports the claim that the vigour of the emerging seedlings reduces with increased seed age, making them less competitive.

Viability (%) determined by the tetrazolium test was higher (*P_50_* = 31.3 days) than that recorded through the germination test, suggesting the seeds may have short-term persistence in the seedbank (between 1 and 3 years). Tetrazolium tests can overestimate the viability (%) of seeds when compared to germination (%), but this is often by a margin of 5% [56]. The seeds tested via the germination test could have demonstrated low germination (%) as a result of stress caused from the artificial ageing treatment. Exposure to a high constant temperature, such as that experienced in the artificial ageing treatment, has been observed to induce secondary dormancy in *N. trichotoma* seeds [26], and therefore, the tetrazolium test may have accounted for these dormant seeds. Secondary dormancy is a protective strategy for mature, hydrated seeds, which is induced by prolonged exposure to unfavourable environmental cues [57,58]. Secondary dormancy has proven difficult to break for other species such as *Coreopsis lanceolata*, even after optimal temperature and light cues return [59].

### 4.2. Seedbank Persistence

The ability for an exotic plant species to develop dense and persistent soil seedbanks contributes to its invasive potential [60]. The ability for seeds to persist for over one year is an important bet-hedging strategy to protect the plant’s population from various disturbance events and unsuitable conditions for germination [61]. Further, having seeds of multiple ages within a seedbank can enhance the genetic diversity within the population [62]. Consequently, these factors make the management of invasive plants with persistent seedbanks complex, time- and cost-consuming, and often, the weeds return after the invaded areas have been treated [63].

Whilst the literature suggests that *N. trichotoma* is able to establish short-term persistent seedbanks (up to three years), it is found, however, that most of the seeds germinate within the first autumn following seed set [24,26], and seedbank density has been observed to be reduced by approximately 40% between March and August [24]. The results of this research support this claim, as only 9% of the seeds buried in May and <1% of the seeds buried in January maintained viability after one year. A similar figure was observed after six months of burial, with only 12.5% and < 1% of the seeds buried in May and January, respectively, being intact and viable. The literature also observes that the germination for *N. trichotoma* predominantly occurs in autumn and winter [35,41], and it was observed in the present study that field germination (%) was higher within the first six months (equivalent to autumn and winter) for the seeds buried in January compared to those buried in May, which initially experienced winter and spring conditions.

The average monthly temperatures observed during this experiment were within the ideal range for germination, ranging from a maximum average daily temperature of 25 °C in the summer months and 14 °C in the winter months [42]. Rainfall was consistent over the 18-month period, with exceptionally high rainfall observed in January 2021 at both sites. Despite a high soil moisture content previously being determined as one of the most important factors for initiating *N. trichotoma* germination [26,42], no variation in in-field germination (%) was observed as a result of the rainfall event, nor was there any significant variation between the two sites despite the Pentland Hills site receiving higher rainfall. The variation in soil type between the two sites may have contributed to seed decay (%), as clay soils observed at Mambourin can hold higher water content compared to the loam soil types observed at Pentland Hills.

A longevity study conducted in New Zealand found a similar proportion of seed decay (%) for *N. trichotoma*, whereby 90% of the seeds were recorded as decayed after 19 months of in-field burial [64]. The presence of an intact cotyledon or radical was required for seeds to be classified as germinated under field conditions. As the burial time increased, the ability to detect these structures prior to their deterioration reduced, and it is likely that the majority of the seeds classified as decayed did, in fact, germinate under field conditions. Further, the seeds considered as germinated under field conditions are likely to have done so recently for these structures to remain intact [65].

Our analysis of the artificial ageing data suggested that a small proportion of the seeds would exhibit long-term persistence, which agrees with other accounts which have identified small quantities of *N. trichotoma* seeds capable of persisting for up to 15 years under field conditions [44,66]. After 18 months of burial, 1.2% of the seeds in both the January and May burial periods remained viable. As *N. trichotoma* can produce as many as 140,000 seeds per plant annually, and seedbank densities have been recorded as high as 50,000 seeds m^−2^ in South Africa [38] and Australia [67], and up to 42,000 seeds per m^−2^ in New Zealand [68], even a proportion as low as 1.2% of the seeds demonstrating persistence could equate to a significant number of germinants that would require ongoing management.

### 4.3. Implications for Management

Understanding longevity can assist with understanding persistence potential, which is a critical element for the effective development of management policies and control strategies. The use of the artificial ageing technique provided an effective method for rapidly estimating the seedbank longevity for *N. trichotoma*, and these results complimented the data obtained by the burial treatment. Although the seedbank persistence categories currently associated with the artificial ageing technique are quite broad, this technique offers a simplistic tool to assist in the development of weed management programs. As a basis, knowing if a species can produce transient, short- or long-term persistent seedbanks can allow managers to make decisions regarding a plant species invasive potential [51]. This research suggests that a high proportion of the seedbank either germinates or decays within the first six months of seed set, regardless of what time of year the seeds are dropped. Therefore, control methods should be implemented in late winter to early spring to maximise control strategies targeting the emerging seedlings, as the majority of the seedbank will have either germinated by this point, or have decayed.

It is suggested that achieving local eradication is more feasible for invasive species that produce transient seedbanks [61], yet *N. trichotoma* is considered very difficult to control and remains widespread at a landscape scale [36,37]. In order for *N. trichotoma* to remain competitive, it must rely on producing high volumes of seeds annually. These seeds are able to travel great distances and recolonise previously treated areas. This indicates the importance for community groups to organise a collaborative, land-scale management for *N. trichotoma*, rather than land managers treating only their own property. This research shows promise in that the seedbank density can be significantly reduced after one year by implementing strategies that prevent seed set.

## 5. Conclusions

*Nassella trichotoma* has a transient to short-term persistent seedbank, with less than 10% of the seeds demonstrating viability after 12 months. After six months of burial, 75 to 83% of the seeds had decayed, and a further 13 to 16% had evidence of in-field germination. Although this species is able to produce exceptionally high seedbank densities, our results demonstrate that to maintain its viability, the species requires continuous seed input annually. The difference in viability observed in the germination trials and the tetrazolium test could indicate secondary dormancy under unfavourable environmental conditions. For this reason, it is recommended that the seedbank should be monitored for at least three years and efforts to prevent seed set and seed migration into treated areas should be prioritised by land managers.

## Figures and Tables

**Table 1:** The effect of increased exposure time to artificial ageing conditions on *N. trichotoma* seeds. The table shows the mean and standard error for germination (%), germination energy (%), germination rate index, and mean germination time at each duration of exposure to the treatment. As no germination was observed for 75, 100, and 120 days under artificial ageing conditions, these results were not included.

Artificial Ageing Time (Days)	Germination %	Germination Energy %	Germination Rate Index	Mean Germination Time
1	72.67±7.62	52.67±6.65	3.76±0.46	23.64±0.40
2	63.33±4.89	33.33±3.21	3.11±0.28	24.02±0.51
5	56.67±5.6	32±3.27	2.34±0.25	25.01±0.28
9	66±5.34	57.33±5.33	3.96±0.34	22.87±0.32
20	28±8.13	25.33±6.08	1.33±0.32	24.59±0.49
30	30.67±9.78	22.67±7.13	1.25±0.36	24.76±1.04
50	22.67±6.59	0	0.36±0.1	30.25±0.62

**Table 2:** Effect of burial depth and time on *N. trichotoma* seed persistence for seeds buried in January and May.

	Time of Burial									
	6 Months		9 Months		12 Months		15 Months		18 Months	
Burial depth (cm)	Germination (%)									
	Jan	May	Jan	May	Jan	May	Jan	May	Jan	May
0	0	11.2	0	10	0	1.6	2	0	2	3.20
1	0	12	3.2	15.2	0	14.8	0.4	5.2	0.4	0.40
2	0	14	0.8	5.2	0	11.2	0.4	9.2	0.4	0.40
4	0.4	12.8	1.6	12	0.8	7.6	6.4	6.8	6.4	0.40
Mean	0.1	12.5	1.4	10.6	0.2	8.8	2.3	5.3	1.2	1.2
SEm±	1.08	1.26	1.46	1.51	2.15	2.07	2.53	2.24	3.12	2.92
Burial depth (cm)	Seed decay (%)									
	Jan	May	Jan	May	Jan	May	Jan	May	Jan	May
0	80.4	74.8	87.2	76.8	88.8	72.4	92	70.8	66.4	78.4
1	80.8	78.4	88.8	77.2	57.6	63.2	77.6	57.6	81.2	79.2
2	77.6	74	90.4	82.4	86.4	76.4	84.4	66.8	82.8	82.4
4	94.8	72	93.2	83.2	96.8	69.6	80.4	74	78.8	86.8
Mean	83.4	74.8	89.9	79.9	82.4	70.4	83.6	67.3	77.3	81.7
SEm±	14.39	12.60	14.80	13.00	14.21	10.84	12.72	9.87	11.14	11.71
Burial depth (cm)	Field germination (%)									
	Jan	May	Jan	May	Jan	May	Jan	May	Jan	May
0	19.6	14	12.8	13.2	11.2	26	6	29.2	30.4	20.4
1	19.2	9.6	8	7.6	42.4	22	22	37.2	18.4	19.2
2	22.4	12	8.8	12.4	13.6	12.4	15.2	24	16.8	15.2
4	4.8	15.2	5.2	4.8	2.4	22.8	13.2	19.2	20.8	10
Mean	16.5	12.7	8.7	9.5	17.4	20.8	14.1	27.4	21.6	16.2
SEm±	3.4	1.5	1.11	1.41	6.21	2.62	2.33	3.54	2.25	1.69

SEm± refers to the standard error of the mean.

## Data Availability

The data that support this study will be shared upon reasonable request to the corresponding author.
